# A national cross-sectional survey of dental anxiety in the French adult population

**DOI:** 10.1186/1472-6831-7-12

**Published:** 2007-10-10

**Authors:** Emmanuel Nicolas, Valérie Collado, Denise Faulks, Brigitte Bullier, Martine Hennequin

**Affiliations:** 1 CHU Clermont-Ferrand, Service d'Odontologie, Hôtel-Dieu, F-63000 Clermont-Ferrand, France; 2 Univ Clermont1, EA 3847, Faculté d'Odontologie, 11 bvd Charles de Gaulle, F-63000 Clermont-Ferrand, France

## Abstract

**Background:**

Dental anxiety is a public health problem but no epidemiological study has been undertaken in France to evaluate its prevalence. The aim of this study was to estimate the prevalence, severity and associations of dental anxiety in a sample of the French adult population.

**Methods:**

A convenience sample of 2725 adults (mean age = 47 years, SD16, minimum = 16, maximum = 101 years), representative of the French population with regard to age and urban distribution, completed a French version of the Corah Dental Anxiety scale (DAS) and a questionnaire relating to their dental appointments.

**Results:**

Moderate dental anxiety (14≥DAS≥13) was revealed for 172 persons (6.2%), while 195 (7.3%) had severe dental anxiety (DAS≥15), giving an overall prevalence of dental anxiety of 13.5%. Prevalence was lower proportionally with age (P < 0.001) and was higher in French overseas territories and in the countryside (P < 0.01). Farmers and low skilled workers were significantly more anxious than executives and shopkeepers (P < 0.001). Anxiety was associated with avoidance of care (p < 0.001) and lack of regular dental appointments (p < 0.001).

**Conclusion:**

Dental anxiety in France appears to concern a similar proportion of the population as in other industrialised European, Australasian or North American countries. Recommendations for prevention and management of dental anxiety are made with reference to dental education and health care services in France.

## Background

Dental anxiety partially limits, or completely prevents, utilisation of oral health care services [1,2]. It increases the prevalence of dental disease [2,3]. Anxious persons present more damaged or missing teeth and less restored teeth [4]. Regular and conventional care is bypassed by dentally anxious persons, who rely on self-care, use of emergency services, and occasionally use of traditional or parallel remedies to relieve pain. The oral health and quality of life of this population are affected [5]. When professional care is provided, it is often given under general anaesthesia without consideration for the aetiological factors behind dental fear. Ideally, the management of patients with dental anxiety requires psycho-behavioural and sedation procedures [6,7] as alternatives to general anaesthesia. Such techniques have been shown to improve patient capacity to cope with dental care over time [8]. These techniques, however, are not always included in undergraduate or postgraduate teaching in France.

The prevalence of dental anxiety has been shown to range between 4 and 20% in the general population of industrialised countries [9-11]. However, there are no available data for France. It is impossible to advocate for services for persons with dental anxiety without an idea of the numbers and types of persons affected. Special care for people with dental anxiety or phobia has a cost [12] and research studies are needed to support reorganisation of both dental teaching and dental services. This study aims to evaluate the prevalence, severity and associations of dental anxiety declared in a large sample of the French adult population and to analyse the impact of psychosocial variables on this anxiety.

## Methods

### Participants

The survey was conducted over a one year period (from May 2004 to May 2005) in collaboration with the French branch of the Soroptimist International association, a group that supports projects to advance human rights. Five thousand anonymous questionnaires were sent to the local branches of the French association. Each member of the association was asked to propose the survey to his/her family members and/or friends over 16 years of age. The association has 49 branches distributed in 31 administrative departments and overseas territories. After completion, the questionnaires were gathered locally and returned by mail to the centre responsible for data analysis.

### Questionnaire

The questionnaire consisted of 4 parts. The first part contained information about the study and a request for consent for participation. The second part collected data relating to demographic data, age, occupational category and place of residence. The third part included the French version of the Dental Anxiety Scale (DAS) [13]. The English language version was translated by three French-speaking and three English-speaking dental experts, and the translation was proofread and validated by one English and one French expert. The four items of the DAS scale were scored from 1 to 5 and summed to give an overall score of 4 to 20. The level of anxiety was evaluated according to Corah [14]: The patient was considered as not dentally anxious for a DAS score<12, dentally anxious for a DAS score reaching 13 or 14, or severely anxious for a DAS score ≥15. The fourth and final part of the questionnaire asked about conditions of utilisation of oral health care services, including date of last appointment, number of dental appointments since childhood, and two questions concerning avoidance of care: "Has dental fear ever delayed or prevented you from making an appointment?" and "Has dental fear ever led you to cancel an appointment?" The possible replies were "yes" or "no".

### Data analysis

Statistical analysis was performed using SPSS^® ^^11.5 ^software. The demographic data (age group, occupational category and residence place) of the study group and those of the French population [15,16] were compared using a Pearson chi-squared test. Descriptive analysis of the results was performed for DAS overall score, avoidance of dental treatment and recall appointments. The influence of place of residence, occupational category, age, number of dental appointments and last dental appointment on the DAS score was performed using a Student Newman Keuls test post Anova (SNK, α = 0.05). Multivariate analysis was performed (3 ways ANOVA) between mean DAS scores and the fixed factors of age, socio-economic status or place of residence. A t-test with a significance level of p < 0.05 was used to compare DAS means scores for avoidance of making appointments and cancellation of appointments. Relationships between the avoidance of care and the estimated number of dental appointments since childhood, the urban distribution, and the occupational category were studied respectively using a Pearson Chi Squared test. Relationships between the number of dental appointments and both the urban distribution and the occupational category was evaluated in the same way.

## Results

**Figure 1:**
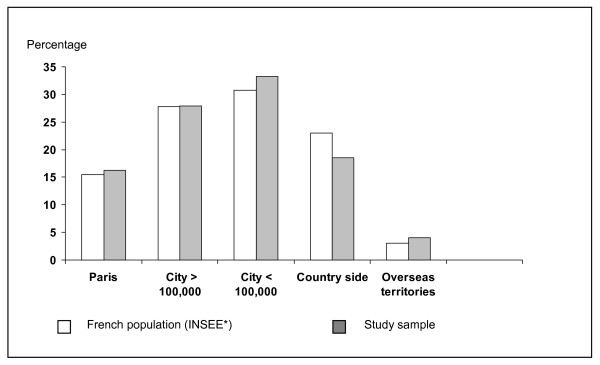
Urban distribution (percentage) of the study population and the French population. No difference was shown between groups (Chi square). City > 100,000 = City > 100,000 inhabitants. City < 100,000 = City < 100,000 inhabitants. (*INSEE: National Institute for Statistics and Economic. 1999).

**Figure 2:**
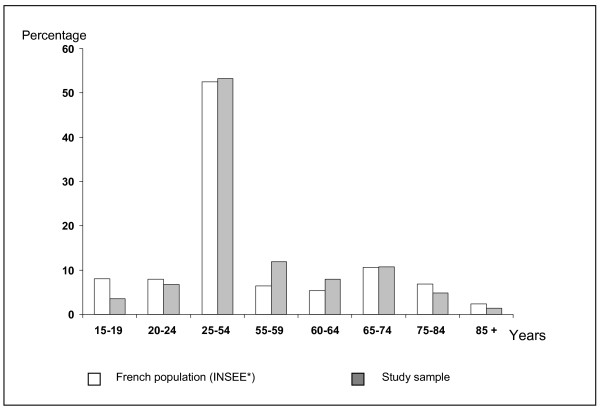
Age distribution (percentage) of the study population and the French population. No difference was shown between groups (Chi square). (*INSEE: National Institute for Statistics and Economic. 2003).

**Figure 3:**
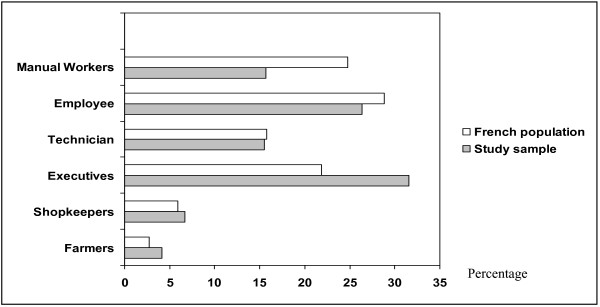
Distribution of occupational category (percentage) of the study population and the French population. Significant difference was shown between groups (p = 0.052. Chi square). (*INSEE: National Institute for Statistics and Economic. 2004).

2725 questionnaires were received and analysed, giving a response rate of 54.5%. There was no difference between the study group and the French population for age distribution (mean: 47.7, SD 16.9 years; min = 16, max = 101) (Figure 1) and the place of residence (Figure 2). A difference was found for the occupational category (p = 0.05) as there were more executives in the study group than in the general population (Figure 3).

Analysis of DAS scores (mean: 8.3 SD 3.48, IC95% [8.15/8.41]) revealed that 86.5 % of the participants did not experience dental anxiety (Score <13, N = 2358, mean: 7.2 SD 2.25, IC95% [7.13/7.31]), whilst 6.2 % were anxious (13≤Score ≤14, N = 172, mean: 13.4 SD 0.49, IC95% [13.33/13.43]) and 7.3 % were severely anxious (Score ≥15, N = 195, mean: 16.6 SD 1.70, IC95% [16.32/16.56]). This gives prevalence for dental anxiety of 13.5% for the participants in this study. Mean values of the DAS scores calculated for urban distribution, occupational category, group of age, dental follow up and avoidance of care were reported in Table 1. The mean DAS scores were lower in older age groups (F = 11, P < 0.001). Dental anxiety was seen to be greater for people living in French overseas territories and in the countryside (SNK, F = 4, P < 0.01) than for those living in the city. Farmers and manual workers were more dentally anxious than executives, technicians, employees and shopkeepers (SNK, F = 8, P < 0.001). The results of the one-way ANOVA analysis were not modified by multivariate analysis, showing that there was no inter-relationship between age, occupation and place of residence on DAS score. Mean dental anxiety scores were higher with avoidance of appointments (t-test, p < 0.001), with cancelled appointments (t-test, p < 0.001) and were lower if the last appointment was within the last 3 years (SNK, F = 13, p < 0.001), and with increased number of appointments (SNK, F = 5, P < 0.01).

**Table 1:** Mean DAS scores by sociodemographic and dental service-use characteristics

variables	Items	Description		DAS score		DAS score/variables
		
		N	%	Mean	SD	p values
site	countryside	451	17	8.4	3.4	p < 0.01
	city > 100,000 inhabitants	792	29	7.9	3.2	
	city < 100,000 inhabitants	939	34	8.3	3.6	
	Paris	420	15	8.6	3.8	
	overseas territories	123	5	9.3	4.1	

age (years)	15–19	99	4	8.5	3.5	p < 0.001
	20–24	190	7	8.6	3.3	
	25–54	1425	52	8.7	3.6	
	55–59	327	12	7.7	3.3	
	60–64	220	8	7.9	3.3	
	65–74	284	10	7.7	3.1	
	75–84	134	5	7.3	2.9	
	85 and more	46	2	6.6	3.1	

number of dental appointments	more than 50	464	21	7.9	3.3	p < 0.01
	20–50	1509	68	8.6	3.5	
	oct-20	131	6	9.7	3.8	
	01-oct	59	3	9.0	4.4	
	never	31	2	10.1	4.5	

last dental appointment (no. of years)	more than 10	45	2	10.2	5.0	p < 0.001
	05-oct	274	10	8.9	3.7	
	03-mai	531	19	8.3	3.4	
	01-mars	1024	38	8.2	3.4	
	within the year	851	31	8.1	3.5	

occupational category	farmers	95	3	9.0	4.2	p < 0.001
	manual workers	152	6	8.0	3.6	
	technicians	840	31	7.8	3.2	
	employees	604	22	8.7	3.7	
	shopkeepers	246	9	8.4	3.6	
	executives	358	13	9.0	3.7	
	retired	430	16	7.2	2.9	

avoidance of care	yes	527	19	11.9	3.9	P < 0.001
	no	2 198	81	7.4	2.8	

cancellation of dental appointments	yes	199	7	12.9	4.3	p < 0.001
	no	2 526	93	7.9	3.1	

Delaying or avoiding making a dental appointment was related to the date of the last appointment (Pearson Chi square, p < 0.01), to the geographic location (Pearson Chi square, p < 0.01) and to the occupational category (Pearson Chi square, p < 0.01) (Table 2). The total number of dental appointments attended by the participants over their lifespan was statistically related to geographic location and to professional status (Pearson Chi square, p < 0.01) (Table 3).

**Table 2:** Dental avoidance by sociodemographic and dental service-use characteristics

Variables		Avoidance of dental appointments				Avoidance of dental appointments/Variables
		
		Yes		No		Pearson Chi Square: p values
			
		N	%	N	%	
Occupational category	farmers	17	17.7	79	82.3	p < 0.001
	manual workers	31	19.9	125	80.1	
	technicians	142	16.7	709	83.3	
	employees	124	20.2	489	79.8	
	shopkeepers	61	24.4	189	75.6	
	executives	92	25.6	267	74.4	

Urban distribution	countryside	97	21.2	361	78.8	p < 0.001
	city > 100,000 inhabitants	139	17.4	658	82.6	
	city < 100,000 inhabitants	188	19.6	771	80.4	
	Paris	79	18.5	349	81.5	
	overseas territories	29	23.6	94	76.4	

Number of dental appointments	more than 50	247	16.2	1 275	83.8	p < 0.001
	20–50	107	22.9	360	77.1	
	oct-20	47	35.9	84	64.1	
	01-oct	18	30.0	42	70.0	
	never	10	29.4	24	70.6	

**Table 3:** Number of dental appointments by occupational category and location

Variables		Number of dental appointments										Number of dental appointments/variables
			
		Never		01–10		10–20		20–50		More than 50		
		
		N	%	N	%	N	%	N	%	N	%	p values
Occupational category	farmers	13	13.5	12	12.5	30	31.3	32	33.3	9	9.4	p < 0.01
	manual workers	2	1.3	12	7.8	23	14.9	59	38.3	58	37.7	
	technicians	0	0.0	54	6.4	119	14.1	326	38.5	347	41.0	
	employees	8	1.3	60	9.8	136	22.3	237	38.9	169	27.7	
	shopkeepers	2	0.8	46	18.5	77	30.9	90	36.1	34	13.7	
	executives	4	1.1	34	9.4	66	18.3	130	36.1	126	35.0	

Urban distribution	countryside	3	0.7	31	6.8	78	17.1	181	39.8	162	35.6	p < 0.01
	city > 100,000 inhabitants	7	0.9	91	11.4	123	15.5	313	39.4	261	32.8	
	city < 100,000 inhabitants	23	2.4	89	9.3	244	25.5	338	35.4	262	27.4	
	Paris	3	0.7	36	8.5	56	13.2	167	39.3	163	38.4	
	overseas territories	0	0.0	31	25.4	35	28.7	37	30.3	19	15.6	

## Discussion

This is the first national study evaluating the prevalence of dental anxiety in France. It gives an estimation of 13.5% of people with moderate or severe dental anxiety within a convenience sample of 2725 participants.

The main weakness of this study is the use of a convenience sample. This limits the degree to which the results can be assumed to represent the French population as a whole. The only difference found between the study group and the French population however, was in occupational category (p = 0.05) as there were more executives in the study group than in the general population (Figure 3). The study design was likely to exclude certain groups known to have a higher prevalence of dental fear, such as children, people with disabilities, elderly persons with dementia and those on the margins of society. This may lead to the hypothesis that dental anxiety was probably underestimated compared to the general population in the current study. Despite these limitations, the estimation is sufficiently high (13.5%) to justify advocacy for new strategies of care for the population with dental anxiety.

These results are similar to those of other industrialised countries in Europe [10], in North America [9], and in Australia [17] (10% to 18%), and is lower than other countries such as China (30%) [18]. In the current study, dental anxiety was related to both longer time since the last dental appointment and greater frequency of cancelled appointments. These results agree with the concept of avoidance of care proposed by Locker [2]. Other authors have associated these behaviours with previous negative experiences [19]. It has also been reported that presence of pain during treatment [20] and the negative attitude or unpleasant remarks of the dentist were correlated with dental anxiety [21].

This study underlines the need for prevention of dental anxiety. Dental anxiety often onsets in childhood [22-24] and young patients should consequently be the target for prevention. It has been shown that early education in children has a positive influence on dental anxiety, improving the long term dental follow-up [25]. This is particularly important as many children already have anxiety at their first contact with the dentist [26]. Although dental anxiety is highly correlated with state anxiety [27], it is often described by patients as an iatrogenic consequence of dental care [28]. This raises the problem of the responsibility of the dental profession and/or of the practitioner. Ethically, if not legally, the aggravation of dental anxiety after a dental episode undertaken without any preventive measures in an anxious patient could possibly be considered as resulting from professional fault. Education should thus be aimed at dental students and professionals. The development of dental anxiety could be prevented with pain control, behaviour management, consideration of the patient as a whole and/or if necessary access to sedation. The inclusion of behavioural sciences in dental education and the integration of ethical considerations in the academic dental curriculum could help to improve the situation [29,30]. For example, French university requirements for the clinical evaluation of dental students are often based on quantitative criteria, such as number of patients treated, or number of teeth restored. Such criteria encourage students to think in terms of quantity but not quality, and are thus incompatible with the principles of avoidance of harm defined by biomedical ethics. This is of particular concern in relation to the treatment of children [31,32]. In addition, conscious sedation is not taught to undergraduates in France.

This study could also be useful to support the development of access centres for persons with dental fear. Deconditionning dental fear needs a multidisciplinary team and is time consuming. Training and rehabilitation seem possible in a favourable environment [33,34]. In Northern Europe [35,36], specific units with multidisciplinary competence and defined protocols allow access to prevention and treatment for anxious patients. In France there are no such teams, although behaviour management and sedation techniques are being developed [8,37]. Moreover, dental anxiety is generally considered as a fatality rather than a disease, despite definition of the different categories of dental anxiety derived from the DSM-IV psychiatric criteria [38]. As a consequence there is no motivation for the development of services. In addition, for the patients able to access one of the few centres treating both dental fear and dental disease, the financial costs of treatment are not recognised by the social security system. This situation increases inequalities in oral health for people with dental anxiety.

## Conclusion

Dental anxiety in France appears to concern a similar proportion of the population as in other industrialised European, Australasian or North American countries. Recommendations for prevention and management of dental anxiety are made with reference to dental education and health care services in France.

## Competing interests

The author(s) declare that they have no competing interests.

## Authors' contributions

EN carried out the analysis and interpretation of the data and wrote the first draft of the manuscript. VC and DF participated in the interpretation of the data, critical review of the draft and in revising the manuscript. BB helped to conceive the study and undertook the acquisition of data. MH conceived and designed the survey and supervised the overall study. All authors have read and approved the final version of the manuscript.

## Pre-publication history

The pre-publication history for this paper can be accessed here:


